# Lattice Dynamics and Electron–Phonon Coupling in Double Perovskite Cs_2_NaFeCl_6_

**DOI:** 10.1021/acs.jpcc.2c07493

**Published:** 2023-01-19

**Authors:** Bin Zhang, Johan Klarbring, Fuxiang Ji, Sergei I. Simak, Igor A. Abrikosov, Feng Gao, Galyna Yu Rudko, Weimin M. Chen, Irina A. Buyanova

**Affiliations:** † Department of Physics, Chemistry and Biology, Linköping University, LinköpingSE-58183, Sweden; ‡ Department of Physics and Astronomy, Uppsala University, UppsalaSE-75120, Sweden

## Abstract

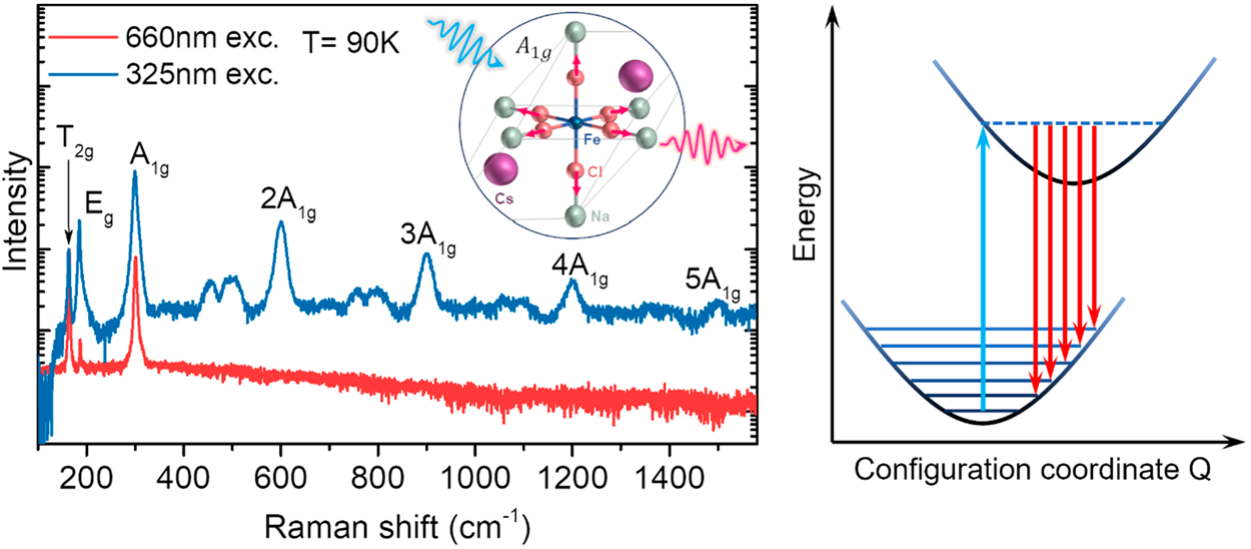

Phonon–phonon and electron/exciton–phonon coupling play a vitally important role in thermal, electronic, as well as optical properties of metal halide perovskites. In this work, we evaluate phonon anharmonicity and coupling between electronic and vibrational excitations in novel double perovskite Cs_2_NaFeCl_6_ single crystals. By employing comprehensive Raman measurements combined with first-principles theoretical calculations, we identify four Raman-active vibrational modes. Polarization properties of these modes imply *Fm*3̅*m* symmetry of the lattice, indicative for on average an ordered distribution of Fe and Na atoms in the lattice. We further show that temperature dependence of the Raman modes, such as changes in the phonon line width and their energies, suggests high phonon anharmonicity, typical for double perovskite materials. Resonant multiphonon Raman scattering reveals the presence of high-lying band states that mediate strong electron–phonon coupling and give rise to intense *nA*_1*g*_ overtones up to the fifth order. Strong electron–phonon coupling in Cs_2_NaFeCl_6_ is also concluded based on the Urbach tail analysis of the absorption coefficient and the calculated Fröhlich coupling constant. Our results, therefore, suggest significant impacts of phonon–phonon and electron–phonon interactions on electronic properties of Cs_2_NaFeCl_6_, important for potential applications of this novel material.

## 1 Introduction

Lead halide perovskites have gained a significant attention over the past decade due to their interesting fundamental physical properties and rapid developments of perovskite-based optoelectronic and photonic devices, such as solar cells and color-tunable light-emitting diodes (LEDs).^1−4^ For example, an external quantum efficiency of perovskite LEDs fabricated through a simple and low-cost solution process has increased to over 20% owing to their exceptional defect tolerance, rivalling commercial LEDs from conventional III–V semiconductors and colloidal quantum dots.^5,6^ Despite the remarkable optoelectronic performance, the toxicity of lead and the lack of stability against prolonged illumination, moisture, and heat in operational environments limit their large-scale deployment.^7,8^ To circumvent these issues inherent to the lead halide perovskites, increasing efforts have been devoted to developing lead-free alternative compounds with better stability and nontoxicity. Here, an emerging and promising strategy is heterovalent substitution of Pb^2+^ with one monovalent (*B*^I^) and one trivalent (*B*^III^) metal cation. This gives rise to a so-called double perovskite architecture with a general stoichiometric formula *A*_2_*B*^I^*B*^III^*X*_6_, where alternating metal cations *B*^I^ and *B*^III^ are octahedrally coordinated by six halide anions *X*, whereas the monovalent cations *A* are embedded in interstitial positions of the octahedral framework. This architecture opens up unprecedented possibilities to engineer structural and electronic properties of these lead-free materials for photonic applications, including solar cells,^9−13^ X-ray detectors,^14^ and warm-white LEDs,^15^ through judicious combination of various *A*-, *B*-, or *X*-site elements and alloying.^16−21^ Moreover, by substituting a metal cation by a magnetic impurity, e.g., a transition metal ion, double perovskite materials may acquire a magnetic response, which is promising for future spintronic applications.^21^

One of the promising lead-free double perovskite materials is the novel Cs_2_NaFeCl_6_ compound. Most recent studies^22^ have shown that this material is thermally stable and exhibits exceptionally strong and completely reversible thermochromism within a wide temperature range that is caused by a strong temperature dependence of its bandgap energy.^22,23^ Since its onset energy of optical absorption lies within the visible spectral range (at around 2.1 eV at room temperature), Cs_2_NaFeCl_6_ could potentially be used in displaying devices, including smart windows, visual thermometers, and thermal sensors. Furthermore, the presence of transition-metal Fe^3+^ ions with an unpaired electron spin suggests interesting magnetic properties attractive for spintronic applications. Exploiting Cs_2_NaFeCl_6_ for practical applications requires a better understanding of its fundamental physical properties, including lattice dynamics and electron–phonon interactions, which are known to be of profound importance for double perovskites but have so far been scarcely studied and explored for this recently synthesized compound. Unlike the conventional inorganic covalent semiconductors (e.g., GaAs), a soft lattice of halide perovskites usually displays large anharmonicity due to phonon–phonon interactions and is also deformable in the presence of charge carriers due to strong electron–phonon interactions.^15,24^ The phonon anharmonicity can shorten phonon lifetimes and cause a finite phonon mean free path, thereby limiting thermal transport performance of the material.^25^ Strong electron–phonon coupling is expected to lead to lattice distortion/displacement that can disturb a periodic potential experienced by charge carriers, giving rise to polaronic effects, broadening, and a Stokes shift of light emission, and also impacts carrier mobility.^26−29^ All of these effects could also be affected by the ordering of B cations in the lattice.^30,31^ Therefore, understanding lattice dynamics and electron–phonon interactions in Cs_2_NaFeCl_6_ is of great importance not only for fundamental insights but also for future optoelectronic and spintronic applications.

In this work, we fabricate high-quality double perovskite Cs_2_NaFeCl_6_ single crystals and examine their vibronic properties and electron–phonon interactions by combining in-depth experimental studies based on polarization-resolved and temperature-dependent Raman and absorption measurements with first-principles calculations.

## 2 Methods

### Crystal Growth

The investigated samples were millimeter-sized Cs_2_NaFeCl_6_ single crystals. For single crystal synthesis, solid CsCl (168.36 mg, 1.00 mmol), NaCl (29.22 mg, 0.5 mmol), and FeCl_3_ (81.1 mg, 0.5 mmol) were dissolved in 7 mL of 37% HCl and then transferred into a 25 cm^3^ Teflon-lined autoclave. The autoclave was sealed, placed in the oven, and then heated to 180 °C for 12 h. After slowly cooling down to room temperature at a rate of 1 °C/h, red Cs_2_NaFeCl_6_ single crystals were formed.

### Optical Measurements

Raman scattering measurements were performed using a confocal Horiba Jobin-Yvon HR800 system in a back scattering geometry. A solid-state 660 nm laser was used as an excitation source during polarization-resolved Raman experiments. To select a desired polarization of the excitation light, a fixed linear polarizer and a rotatable halfwave plate were placed between the light source and surface of the sample, which was mounted in a variable temperature He(N_2_)-flow cryostat. The excitation power was set to be below 1 mW/μm^2^ to avoid heating effects. The induced Raman scattering signal was collected with a long working distance objective (50×, NA = 0.5) and registered by a high-resolution grating (1800/3600 g/mm) monochromator equipped with a charge-coupled device (CCD) detector. Polarization of the scattered light was selected to be either parallel or orthogonal to that of the excitation light by using a halfwave plate coupled with a fixed linear analyzer. For ultraviolet (UV) Raman spectroscopy, the excitation source was switched to a He–Cd 325 nm laser. During transmission measurements, a tungsten halogen lamp was used as a light source, whereas a grating monochromator equipped with a photomultiplier was employed to register the transmitted light.

### First-Principles Simulations

The density functional theory (DFT) simulations were carried out using the Vienna Ab Initio Simulation Package (VASP).^32−34^ We used the PBEsol^35^ exchange-correlation functional and an effective Hubbard *U* correction of *U*_eff_ = 3 eV on the Fe(d) states in the form according to Dudarev et al.^36^ SCPH calculations were employed as implemented in the ALAMODE code.^37−39^ Full details on the methodology and specific settings of both the DFT and phonon calculations are provided in the Supporting Information.

## 3 Experimental Results and Discussion

### 3.1 General Vibrational Properties and Phonon Anharmonicity

**Figure 1:**
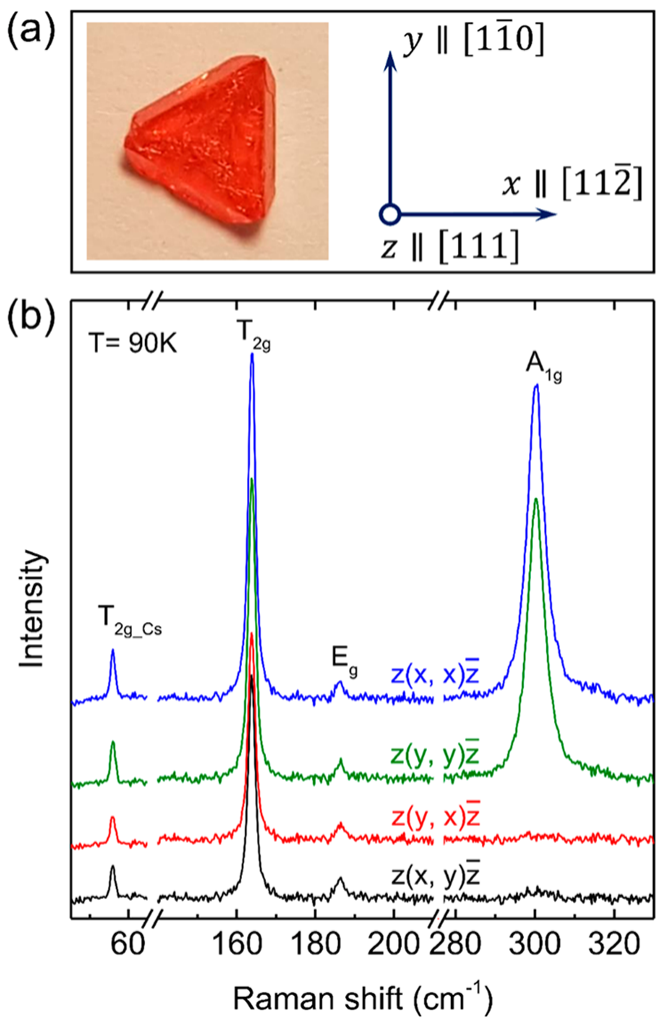
(a) Schematics of the geometry used in polarization-resolved Raman measurements. Left: a top-view optical image of a typical double perovskite Cs_2_NaFeCl_6_ single crystal studied in this work. Right: the utilized Cartesian coordinate system, where *z* axis is chosen to be perpendicular to the top and bottom surfaces of the crystal, whereas *y* axis is oriented along one edge of the top triangular surface. (b) Representative low-temperature (*T* = 90 K) Raman spectra recorded under the 660 nm light excitation. The polarization directions of incident excitation light and Raman scattered light are set according to the corresponding Porto notations. The spectra are offset vertically for clarity.

Figure 1a shows a characteristic top-view optical image of the investigated Cs_2_NaFeCl_6_ double perovskite single crystals (SCs). The crystal exhibits a truncated octahedron geometry typical for this material with top and bottom planes and six sidewall facets belonging to the {111} plane family.^23,40^ The well-defined crystal shape allows us to identify Raman-active phonon modes using polarization-resolved Raman spectroscopy. The experimental configuration for such measurements is shown in the right panel of Figure 1a. The incident light and induced backscattering Raman signal propagate in the direction normal to the top crystal plane, coinciding with the crystallographic [111] direction, whereas the *y* axis is set along one edge of the triangular top facet, i.e., the [11̅0] crystallographic direction. Representative low-temperature (*T* = 90 K) Raman spectra in four polarization configurations, i.e., parallel (*z*(*x*, *x*)*z̅* ↔ *z*(*y*, *y*)*z̅*) and perpendicular (*z*(*x*, *y*)*z̅* ↔ *z*(*y*, *x*)*z̅*) geometries for the polarization vectors of incoming and scattered light, are found to contain several Raman peaks as shown in Figure 1b. Over the temperature range of 80–300 K, the Cs_2_NaFeCl_6_ SC was shown to exhibit a cubic phase. The overall lattice symmetry depends on the ordering of Na and Fe cations and changes from the *Fm*3̅*m* symmetry space group for the perfectly ordered Cs_2_NaFeCl_6_ with alternating [NaCl_6_] and [FeCl_6_] octahedra to *Pm*3̅*m* for their fully random distribution.^41^ A careful group-theory analysis shows that the first-order Raman scattering is forbidden in the latter case, while the zone-center Raman-active modes in the ordered material can be classified as the following irreducible representation: *A*_1*g*_ + *E*_*g*_ + *T*_2*g*_ + *T*_2*g*_*Cs*_. Here *T*_2*g*_*Cs*_ is the external translational mode of the *Cs*^+^ lattice, and *A*_1*g*_, *E*_*g*_, and *T*_2*g*_ are the internal modes of the octahedra.^42,43^ Therefore, the observation of intense Raman scattering involving four phonon modes implies a regular alternating distribution of the Fe and Na atoms in the lattice with the *Fm*3̅*m* symmetry. According to the selection rules for the Raman scattering in the backscattering geometry along the [111] crystal orientation, the *A*_1*g*_ phonon mode is only allowed in the parallel configurations; the *T*_2*g*_ phonon mode has a larger intensity in the parallel geometries with respect to the perpendicular ones; whereas the intensity of the *E*_*g*_ phonon mode is expected to be the same in both parallel and crossed configurations (see also Supporting Information). Experimentally, the mode peaked at 300.3 cm^–1^ only appears in parallel polarization geometries and, therefore, obeys the *A*_1*g*_ symmetry. The feature located at around 186.2 cm^–1^ can be assigned to the *E*_*g*_ phonon mode due to its configuration-independent intensity, whereas the polarization pattern of the two remaining modes indicates their *T*_2*g*_ symmetry. We therefore attribute the high frequency *T*_2*g*_ phonon mode near 163.8 cm^–1^ to bending vibrations of the octahedra cages, whereas the low frequency mode at 56 cm^–1^ is assigned to the translational lattice motion of *Cs*^+^ ions. The suggested mode assignment is further confirmed by the performed density functional theory (DFT) calculations to be discussed below.

**Figure 2:**
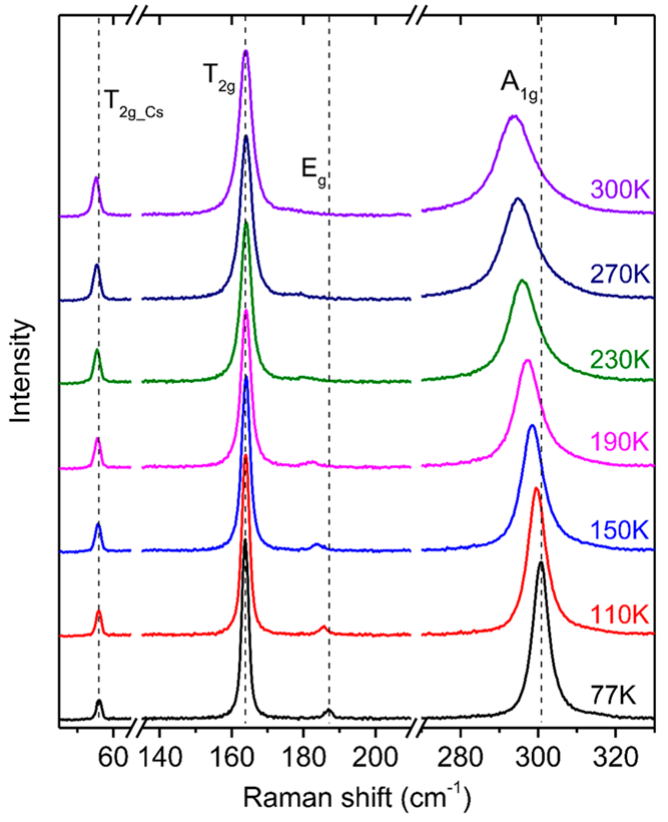
Unpolarized Raman spectra measured at several temperatures ranging from 77 to 300 K. For a better overview, the spectra are offset vertically. The vertical dashed lines are a guide to the eye.

Further information on vibrational properties of the Cs_2_NaFeCl_6_ SCs can be obtained from temperature-dependent Raman measurements. The results of these measurements are summarized in Figure 2. First of all, we note that the four Raman modes studied in this work could be detected without any extra features within the temperature range of 77–300 K, indicating preservation of the *Fm*3̅*m* lattice symmetry and, therefore, the absence of an order–disorder transition up to room temperature. However, substantial broadening of the Raman peaks, which is most pronounced for the *A*_1*g*_ mode, is observed. This is accompanied by a strong temperature-induced red shift of the *A*_1*g*_ and *E*_*g*_ modes, while both *T*_2*g*_ and *T*_2*g*_*Cs*_ modes remain practically fixed in energy.

**Figure 3:**
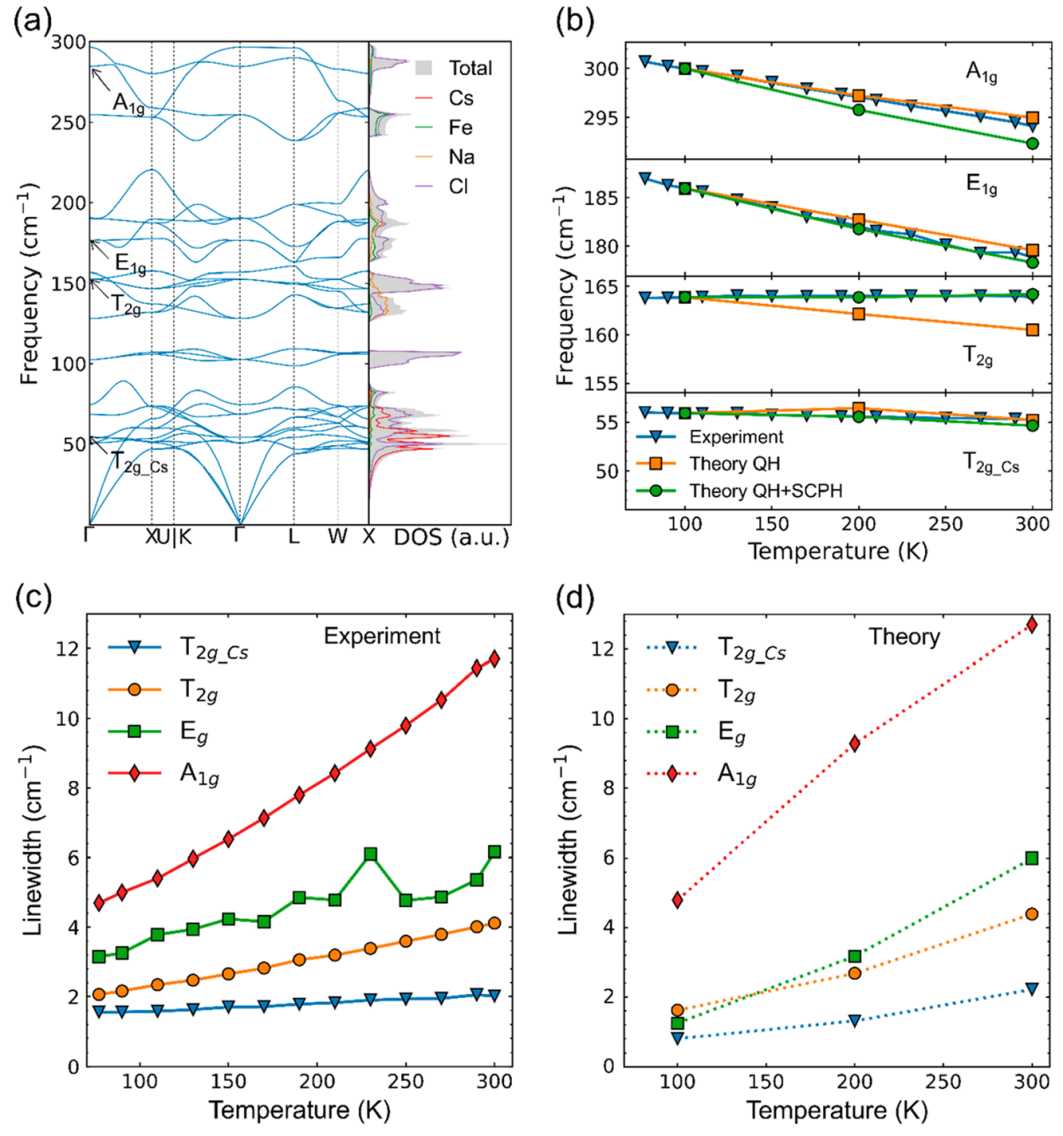
(a) DFT calculated phonon band dispersion curves (the left panel) and total density of states (the right panel). The Raman active modes are marked according to their symmetry. (b) Experimentally measured and theoretically simulated shifts of the phonon frequencies for the four Raman active phonon modes as a function of measurement temperature. The orange squares and green circles are the results of calculations within the QH and QH+SCPH approximations, respectively. The theoretical curves have been shifted to match the measured frequencies at 100 K, in order to compare the temperature dependence more clearly. Measured (c) and calculated (d) line widths of the Raman active modes as a functional of temperature.

Having experimentally identified the symmetry and origin of all active phonon modes, as well as their temperature dependence, we proceed to gain in-depth physical insight into the microscopic origin of the phonon modes by theoretically calculating the vibrational spectrum of Cs_2_NaFeCl_6_. Figure 3a shows the computed phonon dispersion and density of states (DOS) at 100 K. The set of low-energy modes up to ∼85 cm^–1^ corresponds primarily to Cs motion and octahedral tilting vibrations, whereas the modes around ∼100 cm^–1^ and between ∼125 and 150 cm^–1^ are primarily bending and scissoring motions of the octahedra. The modes higher than 150 cm^–1^ correspond to different types of Fe-Cl/Na-Cl stretching motion. The four Raman active modes are indicated in the dispersion, and we note a satisfactory agreement with our experimentally measured frequencies, with a slight underestimation (at most ∼6%) for the high-frequency modes (see Table 1).

**Table 1:** Experimentally Measured Phonon Frequencies (exp.) of the Four Raman Active Modes and Theoretically Expected Phonon Frequencies Calculated by Two Computational Methodologies: the Quasi-Harmonic (QH) Approximation and Self-Consistent Phonon (SCPH) + QH Approximation

	mode frequency (cm^–1^)		
mode (symm.)	exp. (110 K)	QH (100 K)	SCPH + QH(100 K)
*T*_2*g*_*Cs*_	55.9	54.3	54.2
*T*_2*g*_	163.9	150.7	152.6
*E*_*g*_	185.6	177.7	176.4
*A*_1*g*_	299.7	286.1	284.4

Next, we consider the effect of temperature on the Raman-active phonon modes. Figure 3b compares the theoretically deduced frequency shifts with the experimental values (the blue triangles). We show two separate theoretical curves. For the first theoretical curve (see the orange curves), the quasi-harmonic (QH) approximation is applied, where the temperature-induced frequency shift is evaluated only by expanding the volume of the structure, following the measured thermal expansion.^22^ Even though this approximation can reasonably describe the experimentally measured frequency shifts of the *A*_1*g*_, *E*_*g*_, and *T*_2*g*_*Cs*_ modes, it predicts a strong downward shift of the *T*_2*g*_ mode (see the orange squares in Figure 3b), which is not observed experimentally. For the second theoretical curve (see the green curves), we include, in addition to the volumetric thermal expansion, the effect of intrinsic anharmonicity, i.e., phonon–phonon interactions, using a self-consistent phonon (SCPH) methodology^37−39^ (see also Methods and Supporting Information). The corresponding results are shown by the green circles in Figure 3b. Now the good agreement between the experimental and theoretical results is achieved for all Raman-active modes, which shows that explicitly including phonon–phonon interactions is required to qualitatively reproduce the correct temperature-induced variations of the phonon frequencies determined from our measurements.

We now discuss temperature-dependent broadening of the Raman modes. The line width Γ of a phonon mode is linked with the phonon lifetime τ by Γ ∼ 1/τ. In general, there are two primary sources of the phonon damping, i.e., defects/disorder and intrinsic phonon anharmonicity. Phonon lifetime limited by defects/disorder is known to be temperature independent and, therefore, could be responsible for the phonon line width at low temperatures. On the other hand, the intrinsic anharmonicity, that is, decay of a higher-energy phonon into several phonons with low energies, is usually activated with increasing temperature. The measured and calculated line width of the Raman-active modes as a function of temperature is shown in Figure 3c and d, respectively. We note a qualitatively good agreement between theory and experiment. Since the calculated line widths only take into account the intrinsic phonon anharmonicity, this is likely the primary source of the phonon broadening observed in our measurements. This is probably not surprising considering that both single and double halide perovskites are known to be highly anharmonic materials.^24,25^ From Figure 3c is also noticeable that the *A*_1*g*_ line width is significantly broader than that of the other modes at low temperatures and also increases much faster with increasing temperature. This suggests that this mode is more sensitive to phonon–phonon interactions than the other Raman active modes.

### 3.2 Multiphonon Raman Scattering and Electron–Phonon Coupling

**Figure 4:**
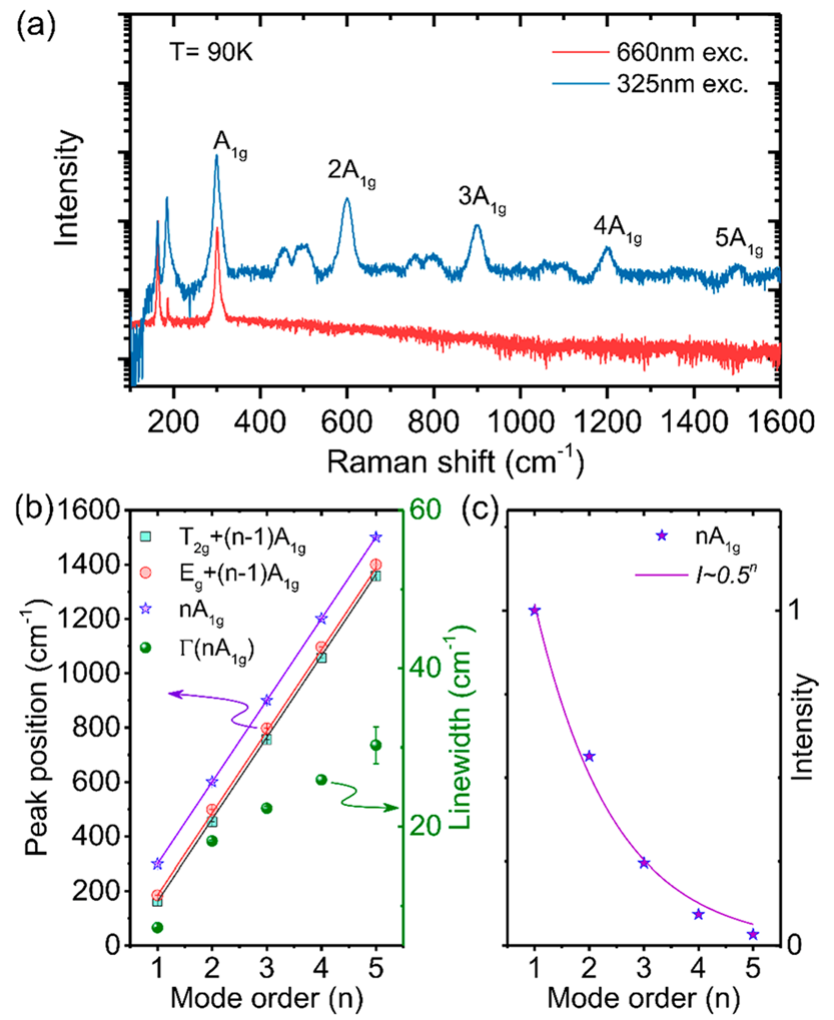
(a) Experimental Raman scattering spectra recorded at *T* = 90 K in a wide frequency range. The blue and red curves represent the spectra measured under the 325 and 660 nm light excitation, respectively. Notations of *nA*_1*g*_ modes label overtones of the *A*_1*g*_ mode in accordance with the mode order *n*. (b) The measured frequency positions (symbols) of the *nA*_1*g*_ overtones and the combination modes of *T*_2*g*_ + (*n* – 1)*A*_1*g*_ and *E*_*g*_ + (*n* – 1)*A*_1*g*_ obtained by fitting the experimental data with multiple Lorentzian functions. The solid lines are linear fits of these data with the same slope of 300.4 cm^–1^. The green filled spheres show the line width of the *A*_1*g*_ overtones as a function of the mode order n. (c) Relative intensities (*I*) of the *nA*_1*g*_ overtones (symbols) deduced from (a). The solid curve is the best fit to the experimental data with the *I* ∼ 0.5^*n*^ function.

Furthermore, our experiments show that additional phonon modes appear in Raman spectra when the excitation photon energy (*h*ω_exc_) is tuned above the Cs_2_NaFeCl_6_ bandgap (*E*_*g*_). Most surprisingly, this effect becomes especially pronounced under the condition of *h*ω_exc_ ≫ *E*_*g*_. This finding is illustrated in the Supporting Information and Figure 4a, which shows a representative Raman spectrum (the blue curve) recorded at 90 K under UV excitation with *h*ω_exc_ = 3.815 eV (or 325 nm). For comparison, we also show a Raman spectrum detected under 660 nm excitation, i.e., under below bandgap excitation with *h*ω_exc_ = 1.879 eV (the red curve). (Note that the bandgap energy of Cs_2_NaFeCl_6_ at 90 K is 2.482 eV.) It is apparent that four additional sets of lines, each containing a strong peak accompanied by two weaker features at its lower-energy side, appear under the UV excitation, suggesting involvement of resonance effects. The frequency and line width of these modes can be extracted by fitting the Raman spectrum with multiple-Lorentzian functions. Since no additional high-energy lattice vibrations are expected from the calculated phonon DOS, these high-frequency modes should originate from higher order phonon processes. The Raman shift of the dominant high-frequency modes is found to display a linear dependence on the mode order *n* with an equal spacing between the neighboring modes of 300.4 cm^–1^, which is very close to the first-order frequency of the *A*_1*g*_ vibrations (see Figure 1b). These modes, therefore, represent a multiphonon progression of the *A*_1*g*_ mode. The two weaker high-frequency features are found to be spaced by multiples of 300.4 cm^–1^ from the first order *E*_*g*_ and *T*_2*g*_ modes and, therefore, stem from the combination modes *E*_*g*_ + (*n* – 1)*A*_1*g*_ and *T*_2*g*_ + (*n* – 1)*A*_1*g*_, respectively. The spacing between the high-order harmonics is very close to the energy of the first-order *A*_1*g*_ mode, which suggests primarily involvement of the zone-center phonons in the multiphonon process. Some contributions from the zone-edge vibrations cannot be ruled out, however, considering flatness of the *A*_1*g*_ dispersion shown in Figure 3a.

The line width of the strong *A*_1*g*_ overtones is found to increase with increasing overtone order, as shown by the green filled spheres in Figure 4b. This suggests that the multiphonons peaks stem from resonant Raman scattering involving real states.^44,45^ Moreover, the overtone intensity (*I*) decreases with increasing mode order following the relation of *I* ∼ *P*^*n*^, which indicates that the *n*-order Raman process is a result of a sequential emission of *n* phonons.^46^ Here *P* denotes the probability of scattering by one *A*_1*g*_ phonon that is determined by electron–phonon interaction. The best fit to the experimental data yields *P* = 0.5 ± 0.03. This value is significantly higher than that characteristic for conventional Raman scattering processes, where the intensity of n-th overtone is expected to be several orders of magnitude lower than that of the (*n* – 1)th overtone due to a weak electron–phonon interaction.^46^ The observation of the intense overtones (up to 5th order!), therefore, suggests strong electron–phonon coupling in Cs_2_NaFeCl_6._

High-order resonant Raman scattering under above bandgap excitation has been observed in resonant Raman spectra of single and double perovskite materials, such as CsPbBr_3_^45^ and Cs_2_Ag_0.4_Na_0.6_InCl_6_,^47^ and discussed in terms of polarons and self-trapped excitons. In this case, the strong electron–phonon interaction can locally deform crystal lattice in the vicinity of a photoexcited carrier leading to its self-trapping. Due to this local distortion, the excited electronic state has a different atomic coordinate than the ground state. If the excitation photon energy is sufficient for an optical transition between the ground and excited states, i.e., higher than the bandgap energy, multiphonon Raman scattering promoted by the Franck–Condon mechanism is activated. In the systems with a large Stokes shift between the ground and excited states, amplitude of the *n*-phonon resonant Raman scattering becomes proportional to the first order of the electron–phonon interaction, greatly enhancing intensities of the phonon overtones.^37,48^ We note, however, that such Raman resonances induced by self-trapped excitons were detected in perovskites when the excitation photon energy was only slightly higher than the bandgap energy.^45,47^ In our case, however, the high-order phonon modes are pronounced when *h*ω_exc_ is equal to 3.815 eV, i.e., exceeds the bandgap energy by about 1.3 eV. This suggests that the monitored resonance involves high-energy conduction- and/or valence-band states that are located well above their respective band edges. We note that the peak position of the Raman resonance could be slightly below 3.815 eV, as follows from the performed temperature-dependent measurements (see the Supporting Information). According to the performed DFT calculations, several high-energy conduction and valence band states with flat dispersion can give rise to resonant light absorption at around 3.815 eV and, therefore, could potentially be involved in the observed Raman resonance (see the Supporting Information). A similar effect was also observed in Cs_2_AgBiBr_6_,^27^ suggesting that it can be common for double perovskites. Though the exact origin of the states involved in the Raman resonance cannot be singled out from the present study, we note that strong multiphonon resonant Raman scattering under above bandgap excitation was also previously observed in LaMnO_3_ and was attributed to the formation of self-trapped excitons that stem from orbital states (the so-called “orbiton”).^37,48^

**Figure 5:**
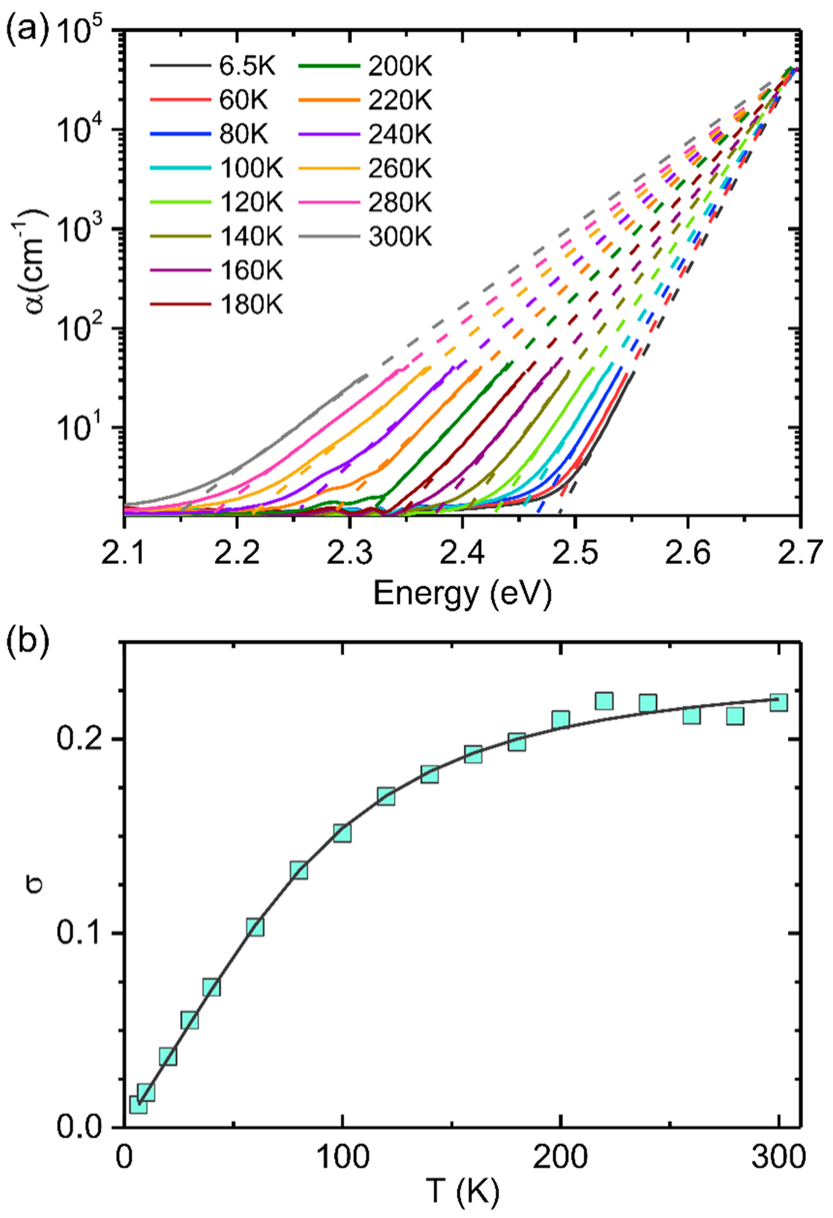
(a) Absorption coefficient measured at various temperatures (solid lines) as a function of photon energy. The dashed lines are expected energy dependences of the absorption tail by the Urbach rule, which converge at a single bundle point at 2.697 eV. (b) Temperature-dependent steepness parameter σ (symbols) extracted from the best fit of the experimental data shown in (a) by eq 1. The solid line represents the best fit of the calculated steepness parameter by eq 2, with σ_0_ = 0.23 and *h*υ_p_ = 0.023 eV.

Further information regarding electron–phonon interactions in Cs_2_NaFeCl_6_ can be obtained by implementing the Urbach analysis^49^ to the temperature-dependent absorption coefficient. It is known that in a wide variety of materials ranging from topologically disordered glasses^50,51^ to pure single crystals,^52^ the optical absorption coefficient α exhibits an exponential tail near the fundamental absorption edge that can be described by the following empirical equation:
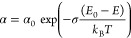
1Here *k*_B_ is the Boltzmann constant, *T* is the measurement temperature, and α_0_ is the absorption coefficient at the convergence energy position *E* = *E*_0_ termed as the Urbach focus. σ denotes the steepness parameter that together with *k*_B_*T* reflects the slope of the Urbach tail on a logarithmic scale. As expected from eq 1, measured spectral dependences of the absorption coefficient in the studied samples at each temperature obey a linear function if displayed in coordinates ln α versus *E*, whereas extrapolations of these dependences to higher energy converge at the same energy *E*_0_ = 2.697 eV, see Figure 5a. By fitting the low-energy tail of the absorption spectra with eq 1, we can then extract the steepness parameter, shown by symbols in Figure 5b. The steepness parameter σ(*T*) varies with temperature as^53,54^
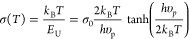
2where *E*_U_ is the Urbach energy and *h*υ_p_ represents the characteristic energy of phonons participating in the formation of the optical absorption edge. σ_0_ is the temperature-independent steepness constant, which is inversely proportional to the strength of exciton–phonon interaction. As shown in Figure 5b, σ(*T*) increases with temperature until the saturation value σ_0_ is reached at high temperatures. The best fit to the steepness parameter σ(*T*) by eq 2 yields *h*υ_p_ = 22.7 meV and σ_0_ = 0.23. Here, the *h*υ_p_ is not equal to any specific phonon energy in the Raman spectrum but is very close to an average value of the phonon energies, suggesting that all phonon modes are involved. Importantly, the magnitude of σ_0_ enables us to evaluate the interaction of charge carriers with lattice vibrations.^26,55^ Following the theory of Toyozawa et al., free exciton becomes spatially localized by the surrounding lattice distortion of its own creation (i.e., a self-induced polaronic potential well) due to strong short-range electron–phonon interaction, if the steepness parameter σ_0_ is below the threshold value σ_c_ = 1.64 for a three-dimensional cubic lattice.^26,56,57^ The extracted steepness constant σ_0_ = 0.23 is apparently smaller than σ_c_, which indicates that the formation of self-trapped excitons and small polarons is likely. We note that this value is even smaller than that of a sister double perovskite Cs_2_AgBiBr_6_(σ_0_ = 0.56),^58^ indicating Cs_2_NaFeCl_6_ possesses a stronger short-range electron phonon interaction. Such strong electron–phonon interaction in Cs_2_NaFeCl_6_ may contribute to the extremely low carrier mobility (μ ∼ 1.06 cm^2^ V^–1^ s^–1^)) in this material, as reported previously.^23^

To further corroborate the presence of small polarons, we calculate the dimensionless Fröhlich coupling constant^59,60^
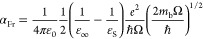
3where ε_∞_ and ε_S_ are the optical and static dielectric constants, respectively; *m*_b_ is the electron band effective mass; Ω is an effective phonon angular frequency; *e*, ℏ, ε_0_ are the electron charge, the reduced Planck constant; and the free space permittivity, respectively. We obtain a value of α_Fr_ = 7.11 for electron polarons, which is much larger than that commonly found for halide perovskites,^61^ especially in comparison with the previously calculated values for other halide double perovskites, e.g., α_Fr_ = 2.54 for Cs_2_AgBiBr_6_,^27^ α_Fr_ = 2.82 for Cs_2_AgBiCl_6_,^62^ and α_Fr_ = 1.99 for Cs_2_AgInCl_6_.^62^ This puts Cs_2_NaFeCl_6_ into the strong polaron coupling regime, where the self-localization of electrons, i.e., the formation of small polarons, is likely. We note that this large value of α_Fr_ is a direct consequence of the large carrier effective mass in the conduction band, which is made up of highly localized bands of primarily Fe(d) character (Figure S4). Indeed, the remaining parameters entering α_Fr_ are comparable to those of the other halide double perovskites mentioned above (see Table S2). We would like to stress that the value of the effective mass and thus the precise value of α_Fr_ is sensitive to the theoretical treatment of the electronic structure of the system, as briefly discussed in the Supporting Information.

## 4 Conclusion

In summary, we have carried out a detailed investigation of phonon–phonon and electron–phonon interactions in highly stable lead-free double perovskite Cs_2_NaFeCl_6_ single crystals by utilizing temperature-dependent Raman and optical absorption spectroscopy. By combining the polarization-resolved Raman spectroscopy and first-principle calculations, the origin and symmetry of four phonon modes active in Raman scattering are clearly identified, that is, one external translational mode of the *Cs*^+^ lattice with *T*_2*g*_ symmetry and three internal modes of the octahedra corresponding to *A*_1*g*_, *E*_*g*_, and *T*_2*g*_ symmetries. We further show that the experimentally observed thermal behavior of the Raman-active modes, such as changes in their line widths and energies, could only be reproduced theoretically by explicitly including phonon anharmonicity, typical for this class of materials. It is also found that intense higher-order Raman scattering modes due to n*A*_1*g*_ overtones (up to the fifth order!) can be detected under above-bandgap excitation with *h*ω_exc_ = 3.815 eV (or 325 nm). This is attributed to a resonant Raman scattering process that involves high-lying band states mediating strong electron–phonon coupling. Strong electron–phonon coupling, which may lead to the formation of small polarons and self-trapped excitons in Cs_2_NaFeCl_6_, is also concluded based on the steepness parameter extracted from a detailed Urbach analysis of the low-energy tail of the absorption spectra measured at different temperatures. This conclusion is further corroborated by the first-principles calculations, which find that the Fröhlich coupling constant in Cs_2_NaFeCl_6_ substantially exceeds that in other double perovskites, due to the large electron effective mass in this material. Our study, therefore, shows that phonon–phonon and electron–phonon interactions play an important role in Cs_2_NaFeCl_6_ and need to be taken into account when analyzing physical properties of this novel material, ranging from electronic and optical properties to thermal properties, key to a variety of future device application areas.

## Supporting Information Available

Raman selection rules in Cs_2_NaFeCl_6_, Urbach tail analysis, and computational details (PDF)
