# Supplementary material for: Lattice Dynamics and Electron–Phonon Coupling in Double Perovskite Cs2NaFeCl6
Source: J Phys Chem C Nanomater Interfaces. 2023 Jan 19;127(4):1908–16. doi: 10.1021/acs.jpcc.2c07493 (PMC9900640; doi:10.1021/acs.jpcc.2c07493)
Supplement: Supplementary file 1 — jp2c07493_si_001.pdf [file jp2c07493_si_001.pdf]

Supporting Information for

Lattice Dynamics and Electron-Phonon Coupling in Double Perovskite $Cs_2NaFeCl_6$

Bin Zhang, Johan Klarbring, Fuxiang Ji, Sergei I. Simak, Igor A. Abrikosov, Feng Gao, Galyna Yu Rudko, Weimin M Chen and Irina A Buyanova**

Department of Physics, Chemistry and Biology, Linköping University, Linköping SE-58183, Sweden.

S1. Raman selection rules in $Cs_2NaFeCl_6$

In polarization-resolved Raman measurements, the observed Raman scattering intensity is determined by

$$I \propto |\hat{e}_s \cdot \boldsymbol{\mathcal{R}} \cdot \hat{e}_i|^2,$$

where $\hat{e}_i$ and $\hat{e}_s$ refer to the polarization vectors of the incident and scattered light, respectively, and $\boldsymbol{\mathcal{R}}$ is the complex second-rank tensor of Raman scattering interaction. The cubic phase of $Cs_2NaFeCl_6$ belongs to the $m\bar{3}m$ point group. In the point group notation, there are four zone-center Raman active modes $(A_{1g} + E_g + 2T_{2g})$. The Raman polarization tensor for each of these modes can be represented as:[1]

$$\mathcal{R}(A_{1g}) = \begin{pmatrix} a & & \\ & a & \\ & & a \end{pmatrix},$$

$$\mathcal{R}_1(E_g) = \begin{pmatrix} b & & \\ & b & \\ & & -2b \end{pmatrix}, \mathcal{R}_2(E_g) = \begin{pmatrix} -3^{1/2}b & & \\ & 3^{1/2}b & \\ & & \end{pmatrix},$$

$$\mathcal{R}_1(T_{2g}) = \begin{pmatrix} & & \\ & & d \\ & d & \end{pmatrix}, \mathcal{R}_2(T_{2g}) = \begin{pmatrix} & & d \\ & & \\ d & & \end{pmatrix}, \mathcal{R}_3(T_{2g}) = \begin{pmatrix} & d & \\ d & & \\ & & \end{pmatrix}$$

In this work, the excitation and scattered beams are directed along the $[111]$ direction, which is orthogonal to the crystal surface, whereas the electric field vector of incoming and backscattering lights lie in the crystal plane. The corresponding polarization vectors for incident and scattered light can be chosen as

$$\hat{e}_i(\hat{e}_s) = \frac{1}{\sqrt{2}}\begin{bmatrix} 1 \\ -1 \\ 0 \end{bmatrix} cos\theta + \frac{1}{\sqrt{6}}\begin{bmatrix} 1 \\ 1 \\ -2 \end{bmatrix} sin\theta = \frac{1}{\sqrt{6}}\begin{bmatrix} \sqrt{3}cos\theta + sin\theta \\ -\sqrt{3}cos\theta + sin\theta \\ -2sin\theta \end{bmatrix},$$

here θ is the angle between $\hat{e}_i(\hat{e}_s)$ and the $[1\bar{1}0]$ axis. Raman scattering intensities under parallel and crossed polarization configurations are summarized in Table S1.

Table S1. The back-scattered Raman intensity in parallel and perpendicular geometries

	**parallel**	**crossed**
$\boldsymbol{A_{1g}}$	a^2	0
$\boldsymbol{E_g}$	b^2	b^2
$\boldsymbol{T_{2g}}$	d^2	$2d^2/3$

S2. Near-bandedge absorption

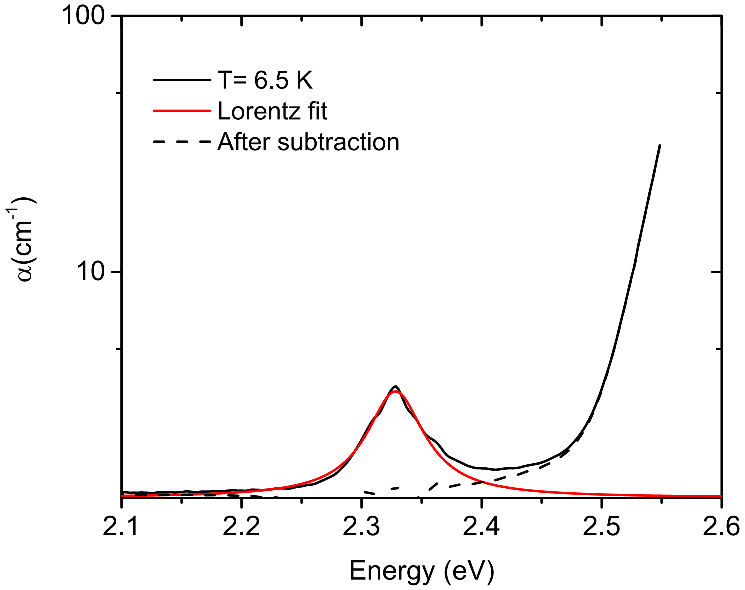

Figure S1. Absorption coefficient measured from a single crystal at T= 6.5 K. The sharp feature was modeled by a Lorentz oscillator, as shown by the red solid line. The black dashed line is the absorption spectrum obtained after subtraction of the sharp feature, which was used for the Urbach tail analysis.

Absorption spectra recorded from $Cs_2NaFeCl_6$ single crystals at low temperatures contain a sharp peak below the bandgap energy – see Fig. S1. The origin of this sharp feature is beyond the scope of this work and is still under investigation. Importantly, the frequency response of this absorption peak I can be well modeled by a Lorentzian function [2]:

$$I \propto \frac{A\Gamma}{4(E-E_0)^2+\Gamma^2},$$

where A, E_0 and Γ represent the amplitude, peak energy and linewidth, respectively. The best fit to the experimental data using this function is shown by the red solid line in Figure S1. Since all parameters describing this absorption peak are found to be temperature-independent, we do not think that it is related to any excitonic transitions responsible for the formation of the absorption edge. It, therefore, was subtracted from the spectra prior to the Urbach tail analysis.

S3. Computational details

All density functional theory (DFT) calculations were performed using the Projector Augmented Wave (PAW) formalism[3], as implemented in the Vienna ab initio simulation package (VASP) [4-6]. We used the PBEsol[7] exchange-correlation functional and an effective Hubbard U correction of U_{eff} = 3 eV on the Fe(d) states, in the form according to Dudarev et al.[8].

Further discussions on how U_{eff} affects the structural, magnetic and electronic properties can be found in Ref. 9. Of relevance for the calculation of the Fröhlich coupling constant, α_{Fr}, in this work is the behavior of the bottom of the conduction band, where increasing the value of U to, say, 6 eV, pushes the t_{2g} band of states above the e_g one. (see Fig. S2 for U=3 eV, where the conduction band edge is the t_{2g} band). Since the e_g band is much more dispersive than the t_{2g} one, this makes the effective mass at the conduction band edge much lower, which in turn reduces the α_{Fr} significantly from $\alpha_{Fr} = 7.11$ for U= 3 eV to $\alpha_{Fr} = 4.86$ for U= 6 eV. We choose to present the value calculated with U=3 eV in the main text as this ordering of the e_g/t_{2g} states agrees with the more expensive hybrid DFT calculations, for mixing parameters at least up to 0.375 [9].

We use a ferromagnetic configuration of the Fe magnetic moments in all calculations since, as is shown in Ref. 9, the effect of different magnetic configurations on relevant properties of $Cs_2NaFeCl_6$ is small.

All phonon calculation were performed using the ALAMODE[10-12] set of codes, using a 2x2x2 (80 atom) supercell. Harmonic (2nd order) interatomic force constants (IFCs) were firsts extracted using the small-displacement method. Non-analytical correction to the dynamical matrix was included. We calculate the dielectric constants ε_∞ and ε_S and Born effective charges using density functional

perturbation theory (DFPT)[13]. Higher-order force constants were then extracted by following the procedure of Ref. 11. Briefly, a set of 60 samples from a low accuracy ab initio molecular dynamics (AIMD) simulation at 400 K are extracted, a random displacement of 0.1 Å is then added to each atom in each snapshot and high-accuracy DFT calculations are performed on the resulting structures. While keeping the 2nd order IFCs fixed, an IFC expansion up to 6th order is then fitted from the force-displacement data using least absolute shrinkage and selection operator (LASSO) regression. A cross-validation procedure was used to choose the regularization parameter. Only 3-body terms for the 4th order and 2-body terms for the 5th and 6th body terms were included in the fit, and a 4.5 Å cutoff were imposed for the 4th-6th order IFCs. The above procedure was repeated for 3 different volumes corresponding to the measured lattice constants at ~ 100, 200 and 300 K. The phonons extracted from the harmonic IFCs at these different volumes are denoted the "quasiharmonic (QH) approximation" in the main text. For all considered cases the resulting residual fitting error was <~ 2.5 %.

The calculations from which the harmonic and higher-order IFCs are extracted used a 620 eV cutoff energy, a convergence criterion of 10^{-8} eV for the self-consistent electronic iterations, a 2x2x2 k-point grid and a Gaussian smearing of 0.05 eV. Atomic positions were relaxed until residual forces were < 10^{-4} eV/Å. The lower accuracy AIMD simulations used a 300 eV cutoff and a 10^{-5} eV convergence criterion. The employed PAW potentials treated the Cs(5s5p6s), Na(2p3s), Fe(3p3d4s) and Cl(3s3p) states as valence.

From the extracted IFCs, renormalized phonon frequencies are obtained using the ALAMODE implementation of the self-consistent phonon (SCPH) theory, with additional inclusion of third order IFC corrections from the "Bubble" self-energy [14].

Mode resolved phonon lifetimes were calculated in the relaxation time approximation (RTA) from three-phonon scattering rates (imaginary part of "Bubble" self-energy) using ALAMODE. From the lifetimes, τ, we extract the phonon linewidths as $\Gamma = 1/(\pi\tau)$, which we directly compare to the linewidths from our Raman measurements. The relevant Brillouin zone integration was performed with the tetrahedron method and a 16x16x16 q-point grid.

The effective phonon frequency ω_{eff} used in the calculation of the Fröhlich coupling constant was extracted by weighting each of the (DFPT obtained) Γ-point phonons modes by the dipole moment they produce, as implemented in the AMSET package.[15]

Table S2. Parameters entering the Fröhlich coupling constant.

Parameter	$Cs_2NaFeCl_6$ (U=3 eV)	$Cs_2NaFeCl_6$ (U=6 eV)	$Cs_2AgInCl_6$[15]	$Cs_2AgBiCl_6$[15]
ε_∞	3.38	3.23	3.97	4.45
ε_S	8.36	7.82	9.7	13.71
ω_{eff} (THz)	4.79	4.88	4.98	4.86
m_b (m_0)	2.37 (t_{2g}-band X --> U)	1.06 (e_g-band X --> Γ)	0.27	0.51
α_{Fr}	7.11	4.86	1.99	2.82

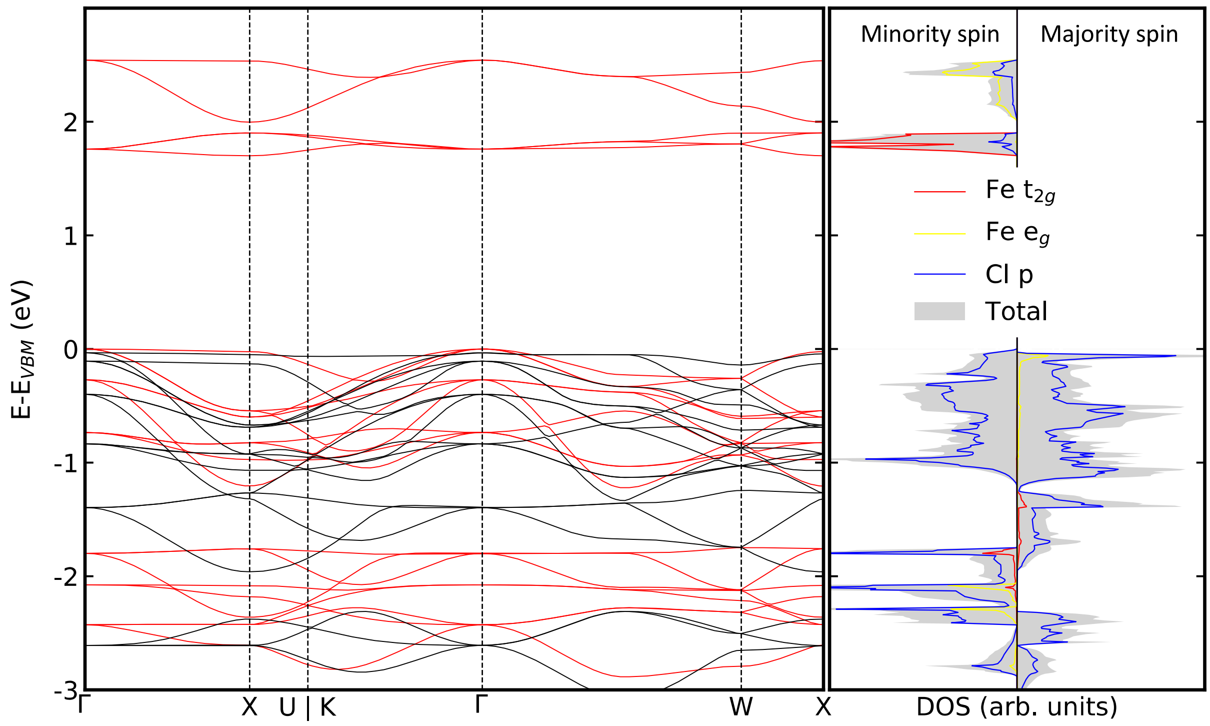

Figure S2. Band structure and density of states (DOS) of $Cs_2NaFeCl_6$ in the ferromagnetic configuration using PBEsol+U(3eV). Majority and minority spin bands are displayed by black and red lines, respectively. Both the band structure and the DOS are aligned with respect to the valence band maximum (VBM).

S4. Temperature and excitation-wavelength dependent Raman spectra.

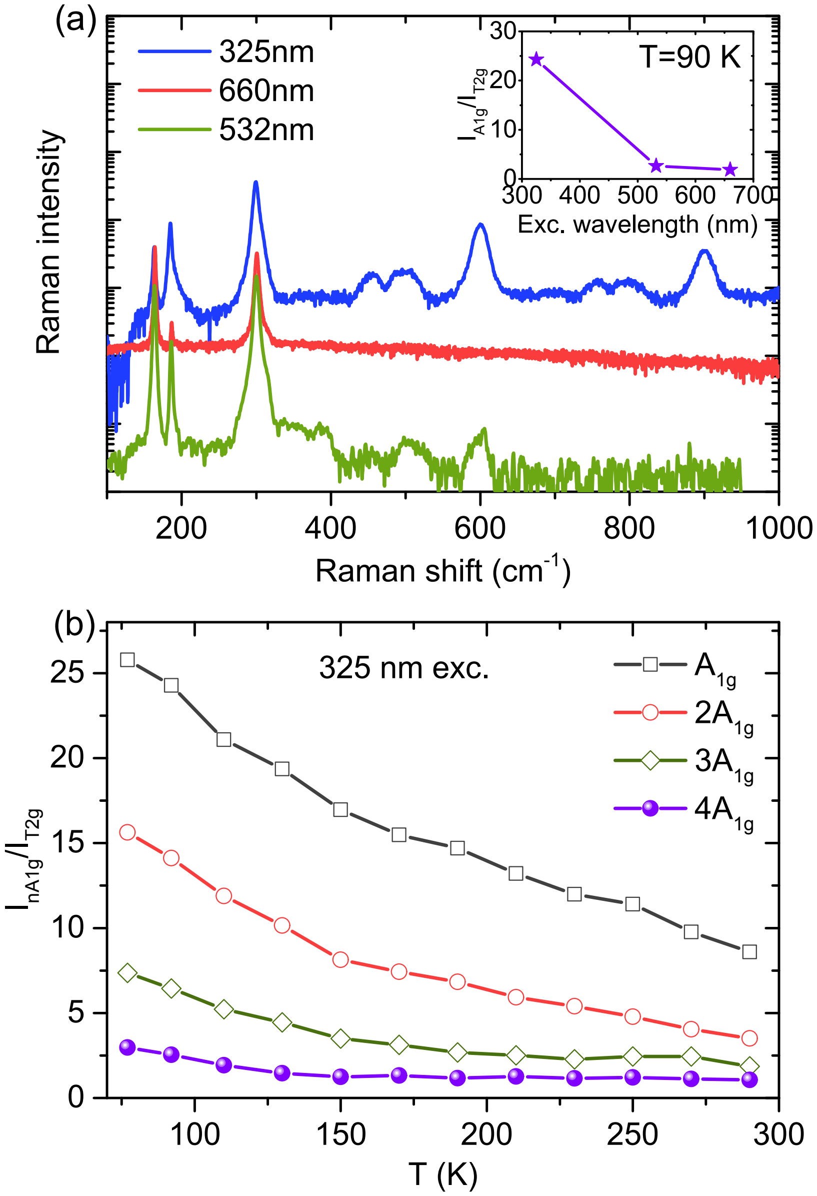

Figure S3. (a) Low-temperature (T= 90K) Raman spectra of $Cs_2NaFeCl_6$ under light excitation at 325 nm (blue), 532 nm (green) and 660 nm (red). The inset shows the intensity ratio of $I_{A_{1g}}/I_{T_{2g}}$ for these excitation wavelengths. (b) The relative Raman scattering intensity $I_{nA_{1g}}/I_{T_{2g}}$ as a function of temperature (T) for the nA_{1g} overtones under the 325-nm light excitation.

In our Raman scattering study of $Cs_2NaFeCl_6$, we used three different excitation light wavelengths, namely, 325 nm, 532 nm and 660 nm – see Figure S3(a). It is found that the intensity of the A_{1g} mode and its overtones becomes substantially enhanced relative to the T_{2g} mode under the 325-nm

light excitation (~3.815eV), suggesting that the excitation photon energy of ~3.815eV is resonant with certain electronic states.

To provide further information on the energy position of the Raman resonance, we performed temperature dependent Raman measurements under the 325-nm excitation. Here, the excitation photon energy was kept constant while the energy position of the involved electronic transition experienced a temperature-dependent blue shift at lower temperatures. As shown in Figure S3(b), the relative intensities $I_{nA_{1g}}/I_{T_{2g}}$ of all nA_{1g} overtones increase with decreasing temperature, indicating that the energy of the electronic transition responsible for the Raman resonance approaches but remains somewhat lower than the excitation energy so that a resonance peak in the intensity profile of $I_{nA_{1g}}/I_{T_{2g}}$ can not be reached by decreasing temperatures. According to the performed DFT calculations, several high-energy conduction and valence band states with flat dispersion could give rise to resonant light absorption at around 3.815 eV and, therefore, could potentially be involved in the observed Raman resonance - see Figure S2.

REFERENCES

1. Cardona, M., *Light Scattering in Solids Ii, Vol. 50 of Topics in Applied Physics*; Springer: Berlin, 1982; Vol. 50.

2. Boyd, R. W., *Nonlinear Optics*, 3rd ed.; Academic Press: London, 2008.

3. Blöchl, P. E., Projector Augmented-Wave Method. *Phys. Rev. B* **1994**, *50*, 17953.

4. Kresse, G.; Furthmüller, J., Efficiency of Ab-Initio Total Energy Calculations for Metals and Semiconductors Using a Plane-Wave Basis Set. *Comp Mater Sci* **1996**, *6*, 15-50.

5. Kozlov, A. N.-E. S. M.; Viñes, F.; Illas, F., Electronic-Structure-Based Chemical Descriptors:(in) Dependence on Self-Interaction and Hartree-Fock Exchange. *Phys. Rev. B* **1996**, *54*, 11169-11186.

6. Kresse, G.; Joubert, D., From Ultrasoft Pseudopotentials to the Projector Augmented-Wave Method. *Phys. Rev. B* **1999**, *59*, 1758.

7. Perdew, J. P.; Ruzsinszky, A.; Csonka, G. I.; Vydrov, O. A.; Scuseria, G. E.; Constantin, L. A.; Zhou, X.; Burke, K., Restoring the Density-Gradient Expansion for Exchange in Solids and Surfaces. *Phys. Rev. Lett.* **2008**, *100*, 136406.

8. Dudarev, S. L.; Botton, G. A.; Savrasov, S. Y.; Humphreys, C.; Sutton, A. P., Electron-Energy-Loss Spectra and the Structural Stability of Nickel Oxide: An LSDA+ U Study. *Phys. Rev. B* **1998**, *57*, 1505.

9. Johan Klarbring, Utkarsh Singh, Igor Abrikosov, Sergey Simak. arXiv:2211.16387 **2022**.

10. Tadano, T.; Gohda, Y.; Tsuneyuki, S., Anharmonic Force Constants Extracted from First-Principles Molecular Dynamics: Applications to Heat Transfer Simulations. *J. Phys.: Condens. Matter* **2014**, *26*, 225402.

11. Tadano, T.; Tsuneyuki, S., Self-Consistent Phonon Calculations of Lattice Dynamical Properties in Cubic $SrTiO_3$ with First-Principles Anharmonic Force Constants. *Phys. Rev. B* **2015**, *92*, 054301.

12. Masuki, R.; Nomoto, T.; Arita, R.; Tadano, T., Anharmonic Grüneisen Theory Based on Self-Consistent Phonon Theory: Impact of Phonon-Phonon Interactions Neglected in the Quasiharmonic Theory. *Phys. Rev. B* **2022**, *105*, 064112.

13. Baroni, S.; De Gironcoli, S.; Dal Corso, A.; Giannozzi, P., Phonons and Related Crystal Properties from Density-Functional Perturbation Theory. *Rev. Mod. Phys.* **2001**, *73*, 515.

14. Oba, Y.; Tadano, T.; Akashi, R.; Tsuneyuki, S., First-Principles Study of Phonon Anharmonicity and Negative Thermal Expansion in ScF_3. *Phys. Rev. Materials* **2019**, *3*, 033601.

15. Ganose, A. M.; Park, J.; Faghaninia, A.; Woods-Robinson, R.; Persson, K. A.; Jain, A., Efficient Calculation of Carrier Scattering Rates from First Principles. *Nat. Commun.* **2021**, *12*, 1-9.
